# 

**Published:** 1890-03

**Authors:** 


### CONTENTS:

Article I.—Non-Metallic Plastic Materials for filling Teeth. By otto Arnold, D.D.S. - - - - - 481
" II.—A Roland for an Oliver. - - - - - 487
" III.—Tenth International Medical Congress, Berlin, 1890 - 496
" IV.—A Bill to regulate the Practice of Medicine in Maryland. - - - - - - 512
" V.—Porcelain Filling. - - - - - - - 516
" VI.—Facial Neuralgia and Allied Neurosis. - - 517
" VII.—Chicago College of Dental Surgery. - - - 518

### COMMUNICATIONS

Missouri State Dental Association. - - - - - 520
Tenth International Medical Congress, Berlin. - - - 521
Twentieth Annual Meeting of the South Carolina State Dental Association. - - - - - - - 521
A Call to Medical Schools. - - - - - - 523
Dislocation Inferior Maxillary. - - - - - - - 524
A Valuable Remedy. - - - - - - - 524

### EDITORIAL.

Well Formed Children. - - - - - - 525

### BIBLIOGRAPHICAL.

Vicks Floral Guide, 1890. - - - - - - - 527

### MONTHLY SUMMARY.

Arsenic. - - - - - - - - - 528
Wnite Chlora-Percha. - - - - - - 528

